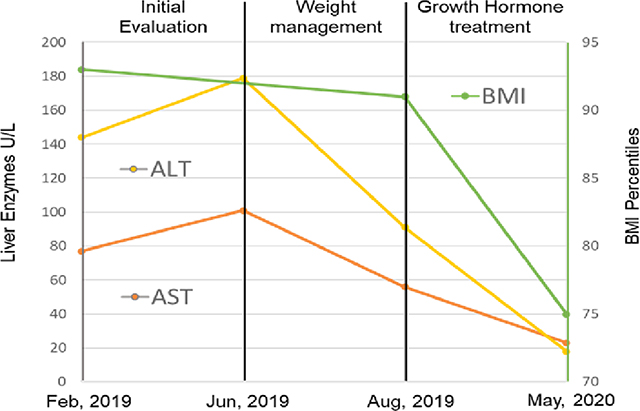# Growth Hormone Treatment Normalized Liver Enzymes in an Adolescent with Obesity and Short Statute

**Published:** 2024-08-07

**Authors:** Mensur Zvekic, Maddie Herbert, Alba Morales, Samir Softic

**Affiliations:** 1 Northern Kentucky University, Department of Anatomy and Physiology, Highland Heights, KY 41099.; 2 Department of Pediatrics, Division of Adolescent Medicine, University of Kentucky College of Medicine, Lexington, KY. 40536.; 3 Department of Pediatrics, Division of Pediatric Endocrinology, University of Kentucky College of Medicine, Lexington, KY. 40536.; 4 Department of Pediatrics, Division of Pediatric Gastroenterology, University of Kentucky College of Medicine, Lexington, KY. 40536.; 5 Department of Pharmacology and Nutritional Sciences, University of Kentucky College of Medicine, Lexington, KY. 40536.

## Abstract

Metabolic Dysfunction Associated Steatotic Liver disease is the most common cause of chronic hepatitis in children and adults. The patients with MASLD have low thyroid hormone activity in the liver. Recent evidence suggests that patients with MASLD may also have haptic growth hormone deficiency. Here, we present a case of a 13-year-old adolescent with obesity and short stature whose liver enzymes normalized with growth hormone therapy. The patient initially presented to the primary care physician’s office, revealing a BMI in the 93rd percentile and elevated liver enzymes (ALT = 170 U/L, AST = 94 U/L). Subsequent visits showed a BMI in the 96th percentile, with further elevation in liver enzymes (ALT = 179 U/L, AST = 101 U/L). Following six months of lifestyle intervention, BMI decreased to the 91st percentile, and liver enzymes improved (ALT = 72 U/L, AST = 56 U/L), but did not normalize. Other causes of chronic hepatitis were excluded. Concurrently, screening for short stature revealed delayed bone age, although insulin-like growth factor 1 (IGF1) and insulin-like growth factor-binding protein 3 (IGFB3) levels were normal. Moreover, the patient failed a growth hormone (GH) stimulation test, revealing GH deficiency, corroborated by MRI findings of pituitary hypoplasia. GH therapy was initiated at pubertal doses. Nine months of GH therapy entirely normalized liver enzymes (ALT = 18, AST = 23), and BMI was reduced to the 75th percentile. GH therapy should be further investigated in adolescents with short stature and MASLD.

## Introduction

Metabolic Dysfunction Associated Steatotic Liver Disease (MASLD) is the most common cause of chronic hepatitis in pediatric and adult subjects. While pediatric patients generally have a mild disease, some may develop more aggressive liver disease characterized by inflammation and fibrosis (MASH) than what is observed in adults. The main treatment options for adolescents with MASH are focused on lifestyle modifications, including increased physical activity and improved dietary habits, such as reduced intake of dietary sugar [1]. Unfortunately for many, these interventions are not sufficient to reverse liver fibrosis. Unlike for adults, there are no FDA-approved therapies for the management of adolescents with severe MASLD and liver fibrosis. Several medications have been tested in adolescents, but they have either proved not to be effective [2,3], or require further testing [4]. Some adolescents have unique risk factors for the development of severe MASLD, such as panhypopituitarism or GH deficiency. In light of no specific pharmacotherapy, identifying and treating unique risk factors for adolescents with severe MASLD holds the promise of improving their liver health.

Growth Hormone (GH), as its name implies, regulates somatic growth in children. Additionally, it exerts diverse and tissue-specific effects on metabolism and energy homeostasis. In the liver, GH regulates hepatic glucose production and lipid metabolism by modulating de novo lipogenesis and triglyceride secretion [5]. In adipose tissue and pancreas, GH contributes to systemic insulin resistance. Therefore, GH deficiency strongly contributes to the development of metabolic syndrome, a key process in MASLD development. Indeed, GH plasma levels are decreased in patients with obesity [6] and MASLD [7]. Moreover, adult subjects with hypopituitarism have an increased risk of MASLD, while GH replacement therapy improves liver enzymes and histology [8]. Here, we describe an adolescent patient with constitutional short stature but normal Insulin-Like Growth Factor 1 (IGF1) and insulin-like growth factor-binding protein 3 (IGFB3) levels. GH replacement therapy resulted in the normalization of liver enzymes and improvement in BMI.

### Case presentation

A 13-year-old male with a medical history significant for ADHD, anxiety, and short stature presented to the primary care physician’s office with a BMI of 93% and was found to have increased liver enzymes (ALT = 170 U/L, AST = 94 U/L). Four months later, a follow-up visit revealed a BMI of 96%, an ALT of 179 U/L, and an AST of 101 U/L. Lifestyle interventions were recommended, and the patient was referred to a Pediatric Gastroenterology clinic for further care. MASLD was diagnosed following an unremarkable comprehensive evaluation that ruled out hepatitis B and C infections, documented normal autoimmune hepatitis profile (anti-smooth muscle antibody, anti-nuclear antibody, liver kidney microsome antibodies, mitochondrial M2 antibody, antineutrophil cytoplasmic antibody, soluble liver antigen antibody and IgG of 1201 mg/dL), A1AT phenotype was M1M2 (level 123 mg/dL), Wilson’s disease was unlikely due to normal ceruloplasmin (31 mg/dL), and screening for celiac disease was negative (TTG IgA 0, total IgA 120 mg/dL). Abdominal ultrasound revealed mild hepatomegaly with diffuse fatty infiltration but no focal lesions.

His growth pattern had consistently followed the 25th percentile until approximately eight years of age, after which it decelerated to the 3rd percentile for age and gender. Simultaneously, he underwent assessment in the Pediatric Endocrinology clinic for short stature. Screening labs for short stature revealed IGF1 and IGFB3 levels (166 ng/mL and 4.5 mg/L, respectively) within the normal range. Hand X-ray documented bone age of 12 years and 1 month. Subsequently, he failed a GH stimulation test with a peak GH of 3.3 (normal > 10.0 ng/L), and a diagnosis of GH deficiency was made. An MRI of the brain showed the pituitary gland was small for age, measuring 3.5 mm in craniocaudal dimension. GH therapy at pubertal doses was initiated. A follow-up in the GI clinic showed complete normalization of liver enzymes (ALT 18 U/L, AST 23 U/L) and a reduction in BMI to 75% (Figure 1). Liver enzymes remained normal (ALT 19, AST 17 U/L) during three years of follow-up, and the patient achieved normal adult height at 73% for age and gender.

## Discussion

Here, we present a case of a 13-year-old male with short stature and MASLD whose liver enzymes normalized following GH replacement therapy. Similar observations were made in an adult patient with growth hormone deficiency, where six months of GH replacement therapy ameliorated MASH and improved lipid profile [9]. These findings are consistent with the increased prevalence of MASLD in patients with pituitary dysfunction compared to age and BMI-matched controls [10].

Moreover, GH levels correlate with intrahepatic lipid content in individuals without disorders of GH secretion. In a study that involved 59 individuals, 16 of whom had increased liver fat content (>5.6%) lower levels of GH and IGF-I were associated with higher liver fat content [11]. Additionally, GH levels during the oral glucose tolerance test were significantly lower in individuals with increased liver fat compared to those without liver disease. ^11^ Others have confirmed that GH action is lower in patients with MASH [12] including significantly lower levels of IGF-1, IGF-2, and the ratio of IGF-1 to IGFBP-3. Though the exact mechanism remains unclear, decreased hepatic GH levels are thought to worsen glucose tolerance by increasing hepatic glucose output while at the same time lowering fatty acid oxidation and triglyceride export from the liver. Additionally, diminished GH activity in obese individuals can trigger metabolic changes in adipose tissue and skeletal muscle, contributing to insulin resistance and impaired glucose tolerance.

In summary, adolescent patients presenting with MASLD and short stature should prompt evaluation of GH action. GH replacement therapy completely normalized liver enzymes in our patient, with sustained improvement after three years of follow-up. On the other hand, untreated pituitary dysfunction has been associated with severe MASLD and increased mortality [13]. Further research is needed to determine the utility of GH treatment in patients with MASLD.

## Figures and Tables

**Table 1:** The left Y-axes show ALT and AST values over time, while the right Y-axes demonstrate patients’ age and sex-specific BMI percentiles.